# A previously unidentified *Chorioptes *species infesting outer ear canals of moose (*Alces alces*): characterization of the mite and the pathology of infestation

**DOI:** 10.1186/1751-0147-49-21

**Published:** 2007-09-10

**Authors:** Gete Hestvik, Monika Zahler-Rinder, Dolores Gavier-Widén, Ronny Lindberg, Roland Mattsson, David Morrison, Set Bornstein

**Affiliations:** 1 Department of Biomedical Sciences and Veterinary Public Health, Swedish University of Agricultural Sciences, Box 7028, 750 07 Uppsala, Sweden; 2 Bavarian Health and Food Safety Authority, Veterinärstrasse 2, D-85764 Oberschleissheim, Germany; 3 Institute for Comparative Tropical Medicine and Parasitology, Ludwig Maximilian University, Leopoldstrasse 5, D-80802 Munich, Germany; 4 Department of Wildlife, Fish and Environment, National Veterinary Institute (SVA), Box 7073, 750 07 Uppsala, Sweden; 5 Department of Parasitology, National Veterinary Institute (SVA), Box 7073, 750 07 Uppsala, Sweden

## Abstract

**Background:**

During the past decade, *Chorioptes *mites occupying the outer ear canals have been a common finding at routine necropsies of moose (*Alces alces*) in Sweden, but neither the taxonomy of the mites nor lesions from the infestation have been investigated. In this study, the mites are characterized by morphological and molecular techniques, and the histopathology of the skin of the outer ear canal is described.

**Methods:**

External auditory meatuses from 53 necropsied moose were examined for the presence of *Chorioptes*, and samples from outer ear canals were taken for histopathological and microbiological examination. A proportion of the mites from each moose was identified to species. The DNA was extracted from mites from three moose, and their ITS-2 sequences were determined; these sequences were compared phylogenetically to sequences from other *Chorioptes *taxa.

**Results:**

*Chorioptes *mites were found in 43 (81%) of the 53 moose. The mites had morphological and genetic characteristics distinct from those of *C. texanus *and *C. bovis*, the two species generally accepted within the genus. Morphology also did not argue for a diagnosis as *C. crewei*, *C. mydaus *or *C. panda*. On histopathology, lesions were characterized by a hyperplastic perivascular to interstitial dermatitis with epidermal hyperkeratosis and crust formation. Dermal inflammatory infiltrates were composed of mixed T- and B-lymphocytes, plasma cells and macrophages, whereas eosinophils were notably uncommon. *Staphylococcus aureus *was grown from the infested epidermis of five of 14 examined moose.

**Conclusion:**

*Chorioptes *mite infestation was frequently detected in the outer ear canals of moose in Sweden. The mites were evidently pathogenic, being associated with inflammatory lesions of the external auditory meatus. Our studies indicate infestations with a previously undescribed *Chorioptes *species.

## Background

Ectoparasites of the genus *Chorioptes *(Acari: Psoroptidae) are distributed worldwide, infesting domestic as well as wild herbivores [1,2]. These non-burrowing mites are commonly found on cattle, sheep, goats, horses and the New World camelids, where they are a common cause of mange and have considerable veterinary importance. The affected skin areas vary with host and degree of infestation, but the extremities or tail regions are commonly involved. The entire life cycle, from egg-laying through larval and nymphal stages to mature mites, takes place on the same host, and spans approximately three weeks [3].

At present, the taxonomy of *Chorioptes *is unclear. Two species, *Chorioptes bovis *(Hering, 1845) and *Chorioptes texanus *Hirst 1924 are generally accepted [1,4], based on morphology and genetic differentiation, while the existence of three further species, *Chorioptes crewei *Lavoipierre 1958, *Chorioptes mydaus *Fain 1975 and *Chorioptes panda *Fain and Leclerc 1975, is still questionable [2,5]. Both *C. bovis *and *C. texanus *are ubiquitous mites with a low degree of host specificity. These mites are mostly found to infest the skin surface of the body, and are rarely found in the ears of the hosts [4,6]. One exception is reindeer (*Rangifer tarandus*), in which *C. texanus *has been considered to be a primarily auricular mite [7]. *C. texanus *has also been isolated from cattle (*Bos taurus*), goats (*Capra hircus*), roe deer (*Capreolus capreolus*), sika deer (*Cervus nippon*) and moose (*Alces alces*) [1,5,8-13]. Reported hosts of *C. bovis *include wild and domestic Bovidae, Cervidae, Equidae and Camelidae [4].

Moose are widely distributed and inhabit almost all rural parts of Sweden. The population amounts to approximately 300,000 during summertime, but is reduced to about 200,000 by hunting in fall [14]. Compilation of diagnoses at routine necropsies at the National Veterinary Institute (SVA) 1986–91 showed various diseases in the Swedish moose population. Among the most frequent diagnoses were traumatic injury, microbial infectious disease, elaphostrongylosis and tumours [15].

Otoacariasis caused by *Chorioptes *was confirmed to occur frequently in Swedish moose found dead or shot in the field and examined at SVA during the last decade [16]. Preliminary studies on the mite indicated a hitherto undescribed taxon of *Chorioptes*. To the best of our knowledge the lesions associated with *Chorioptes *infestation in moose have not been reported. In the present study, the morphological and molecular characteristics of the mite are reported, and the pathology of the infestation in the outer ear canal of moose is described.

## Methods

### Source of material

Moose found dead or shot in the field were submitted to SVA during the period 1997 and 2000–2006, and examined as part of a health monitoring programme. External auditory meatuses from 53 necropsied moose, sampled at convenience, were examined for the presence of *Chorioptes *spp. The degree of post-mortem change in the carcases ranged from mild to marked. Forty-three of the moose originated from seven counties in central Sweden (Stockholm, Uppsala, Sörmland, Örebro, Västmanland, Dalarna and Gävleborg), and 10 from more southerly regions (the counties of Östergötland, Kalmar, Skåne, Halland and Älvsborg). The nutritional state was judged to be normal, subnormal or cachectic (with serous atrophy of fat). The age of the moose was estimated by cementum ageing analysis [17]; and the sex was recorded in 50 animals.

Both outer ear canals of each moose were investigated. The ears with their attached earflaps were cut away as close to the scalp as possible. The ear canals were dissected from attached tissues, and a segment 2–4 cm long of the inner part of the canals was removed. All moose were subjected to parasitological examination, while 28 and 14 were subjected to histopathological and microbiological examination, respectively. No examination for mites was performed on the skin and fur of other body sites.

### Parasitological examination

The ear-canal samples were cut open, exposed to 25–27°C for 30 min, after which the inner surface of the canals was examined under a stereomicroscope. If no mites were observed, skin scrapings were obtained and treated with 10% KOH for 5 hours. After centrifugation, the supernatant was discarded, and a few drops of glycerin were added to the sediment, which was then searched for mites. A proportion of the mites from each moose was identified to species. In 32 moose the level of infestation with mites was subjectively scored: low (n = ≤10), mild (n = >10 – ≤50), moderate (n = >50 – ≤500), or high (n = >500). When degrees of infestation differed between the ears, the scoring was based on the most heavily infested ear, and moose with one ear negative and the other positive were scored according to the positive ear.

### Morphological investigations of mites

Diagnosis of the mites to genus was made according to Fain [18], while *Chorioptes *species differentiation was based on a key given by Fain and Leclerc [19]. Opisthosomal seta 1, 2 and 3 located at the opisthosomal lobes, and seta 4 originating from the caudal body margin between the opisthosomal lobe and leg 4, as well as seta 5 located at tarsus III, were measured in up to 20 male mites isolated from three moose (seta numbering according to Sweatman [7]). For comparison, these setae were also measured in up to 40 male *C. texanus *mites collected from cattle in Germany. Statistical comparisons were done using the Student's *t*-test.

### DNA extraction, polymerase chain reaction and sequencing

Up to 25 mg of each skin scraping from three moose (M289, M290 and M291) was frozen in liquid nitrogen and grounded to a fine powder. DNA was then extracted using the QIAamp DNA mini kit (Qiagen, Hilden, Germany) according to the instructions of the manufacturer, and eluted in 50 *μ*l elution buffer. Primers RIB-4 (CCA TCG ATG TGA A(C,T)T GCA GGA CA) and RIB-3 (CGG GAT CCT TC(A,G) CTC GCC G(C,T)T ACT) were originally designed for the amplification of the second internal transcribed spacer (ITS-2) of the rDNA from *Dermacentor *ticks [20]. Polymerase chain reaction (PCR) amplifications, as well as cloning and sequencing, were done with 5 *μ*l of the DNA solution as described elsewhere [5].

### Molecular sequence analysis

Consensus ITS-2 sequences, here called genotypes, were constructed manually by comparing alignments obtained using the Clustal W (version 1.83) algorithm [21]. Mutations at a given position occurring in only a single clone were classified as polymerase errors, or (indistinguishable from them) as rare genotypes, and were not included in the genotype sequence. Mutation rates were calculated by counting substitutions, deletions and insertions as one mutation each, and dividing the sum by the average length of the sequences. Identities were determined as differences between 100% and the respective mutation rate. To estimate genetic variation between different individuals within a representative skin scraping, and the frequency of polymerase errors and rare polymorphisms, ten clones generated in ten PCR reactions from DNA obtained from moose M290 were sequenced. In the sequences obtained from the other skin scrapings, mutations at sites that had not previously been demonstrated to be polymorphic were verified by at least one other clone originating from an independent PCR amplification.

For comparison, representative ITS-2 sequences obtained from *C. texanus *and *C. bovis *using the same PCR protocol and cloning strategies [5] were included in the analysis [EMBL:EF191362, EMBL:EF191363, EMBL:EF191364, EMBL:EF191369, EMBL:EF191372, EMBL:EF191375], as well as other *Chorioptes *ITS-2 database sequences [EMBL:AF123081, EMBL:AF123082, EMBL:AF123084, EMBL:EF053119, EMBL:EF053120, EMBL:EF053122, EMBL:EF053123]. Sequences from two other members of the Psoroptidae, *Otodectes cynotis *[EMBL:AF367699] and *Psoroptes *[EMBL:EF429269], were used as the outgroup.

The initial multiple sequence alignment was produced using Clustal W, and then manually modified so that it was consistent with the mite ITS-2 secondary-structure model [22]. The fit of the sequences to this model was assessed using the MFold (version 3.2) program [23]. The final alignment had 309 positions for the ITS-2 region of 287 bp (bases 45–331 of [EMBL:EF433564]).

Phylogenetic trees were produced via both maximum parsimony and maximum likelihood algorithms, using the PAUP* (version 4.0b10) package [24]. Maximum-parsimony heuristic searches used 100 random-addition sequences of TBR branch swapping, as well as 20 replications of the parsimony ratchet [25] based on 200 iterations of TBR branch swapping. Maximum-likelihood heuristic searches used the ratchet (Nixon) [26] strategy based on 100 iterations of TBR branch swapping. The GTR+G substitution model was used, determined after preliminary testing with the ModelTest program [27], and the parameter values (fixed during the searches) were estimated using successive approximations [28].

Support for the phylogenies was measured by bootstrapping. For the maximum-parsimony analyses this was based on 2,000 pseudoreplicates, each with 100 random-addition sequences of TBR branch swapping, while for maximum-likelihood analyses it was based on 200 pseudoreplicates, each with 10 random-addition sequences of TBR branch swapping.

### Histopathological examination

Pieces of the sampled inner ear canals were fixed in 10% neutral buffered formaldehyde, embedded in paraffin, and routinely processed for histopathology. Sections cut 4 *μ*m thick were routinely stained with haematoxylin and eosin (HE), and selected sections were stained with periodic acid Schiff (PAS) for aid in identification of parasites, Grocott for fungi, Gram's stain for bacteria, toluidine blue for mast cells, and Lendrum's method for eosinophils [29]. For further characterization of inflammatory cells, skin specimens from five moose were investigated for lymphocyte subsets, employing monoclonal mouse anti-human antibodies CD3 F7.2.38 (Dakocytomation, Glostrup, Denmark) and CD79αcy (Dakocytomation, Glostrup, Denmark) for T-cells and B-cells respectively, in the Dako EnVision +^® ^system.

### Microbiological examination

Smears from pieces of the sampled inner ear canals were plated onto Blood Agar Base (Difco) with 5% horse blood and Blue-agar Base with 1% glucose for bacteriological culture. The plates were incubated at 37°C, and inspected for growth after 24 and 48 hours. For mycological examination, the smears were plated onto 2% Sabouraud glucose agar (Difco) with chloramphenicol. The plates were incubated in an aerobic environment at 27°C, and inspected for a period of 10 days. Isolated strains were recultured, and identified by morphological and physiological characteristics according to standard methods.

## Results

Infested moose were found in all parts of the study area, covering the mid and south regions of Sweden. Of the 53 moose examined parasitologically, 43 (81%) were infested with *Chorioptes *sp. Five of those only had one ear infested. The degree of infestation was scored in 32 moose, in 7 it was low, in 8 mild, in 11 moderate and in 6 moose it was high. Twenty-eight moose had similar degrees of infestation in both ears, while in two moose the degree of infestation between ears varied, and two had only one ear infested.

Of the infested moose, 33 were females and eight were males, whereas the sex was not recorded in two animals. Nine moose showed a subnormal nutritional state, and 21 were cachectic. The 10 non-infested moose included six females, three males and one with the sex not recorded. Four of them had a subnormal nutritional state, and two were cachectic. The age distribution of both infested and non-infested moose ranged from calves to 23 years. As judged from post-mortem records, the panorama of necropsy diagnoses did not differ between infested and non-infested animals, and was similar to that found in the material of Swedish moose necropsied at SVA 1986–1991 [15].

### Morphology of mites

**Figure 1:**
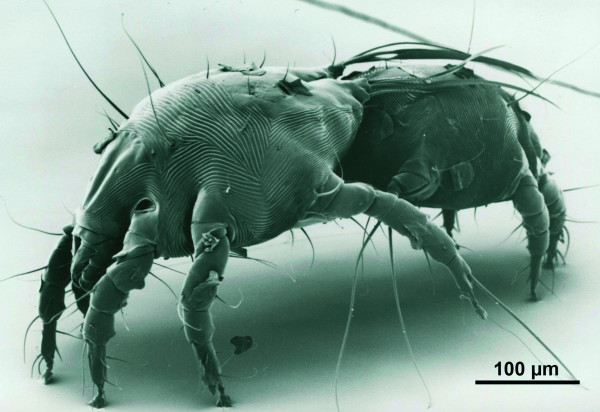
**Scanning electron micrograph of two *Chorioptes *mites (a couple)**. The mites were obtained from the outer ear canal of one of the moose, and their morphology was described in detail in this study.

**Figure 2:**
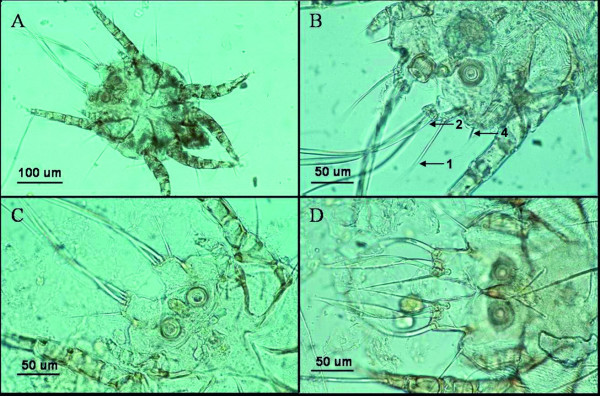
**Light micrographs of individual male *Chorioptes *mites, showing the caudal end with opisthosomal lobes**. **A: ***Chorioptes *sp. sampled from Swedish moose, overview. **B: ***Chorioptes *sp. sampled from moose, details. Measured setae 1, 2 and 4 are indicated by arrows. Seta 3 and seta 5 cannot be seen. **C:***C. texanus *collected from cattle in Gemany. **D: ***C. bovis *sampled from horse in Germany.

The mites were identified as *Chorioptes*, based on males with short pedicles and caruncles (suckers) at legs III and IV, a single long seta at tarsus of legs III, as well as with legs III about twice as long as legs IV (Figure 1 and 2A). The opisthosomal lobes were as long as wide, and bore five setae. Three long and strong setae (setae *l4*, *l5 *and *d5 *according to Fain [18]; seta *l5 *corresponds to seta 2 in figure 2B and table 1) arose, very close to each other, from the apical margin of the lobe. Two of these setae, *l4 *and *d5*, were flattened and blade-like at their apical thirds, while seta *l5 *(seta 2) did not increase in width apically. A fourth seta (seta 1 according to Sweatman [7]; seta *ae *according to Fain [18]) was shorter than the three setae mentioned above, and arose latero-apically at a small accessory lobe forming a small angle with relation to the main lobe. This seta was separated by a distinct gap from the group of the three other setae (Figure 2). The fifth opisthosomal seta was very short and fine. It was found at the medial side of the lobe and arose dorsally.

**Table 1:** Lengths (*μ*m) of the five setae in male *Chorioptes *mites.

No. of seta (location)	*Chorioptes *sp. from moose	*C. texanus *from cattle
	
	mean ± SD range n	mean ± SD range n
1 (at the opisthosomal lobe)	85.3 ± 9.3* 71–106 20	54.2 ± 7.3* 37–69 40
2 (at the opisthosomal lobe)	234 ± 14* 198–251 19	164 ± 8* 145–185 40
3 (at the opisthosomal lobe)	28.8 ± 3.7* 24–40 13	24.3 ± 5.4* 13–32 31
4 (at the caudal body margin)	26.3 ± 2.9 19–32 19	24.9 ± 3.9 16–32 38
5 (at tarsus III)	21.1 ± 5.4 13–32 17	18.3 ± 7.2 8–40 35

n = number of measurements

SD = standard deviation

* differences were significant at *p *< 0.01, based on *t*-test.

Within *Chorioptes*, the mites detected in moose in Sweden were found to have more or less quadrangular opisthosomal lobes, and outer opisthosomal setae *ae *(seta 1) distinctly shorter than setae *l4*, *l5 *(seta 2) and *d5*, which arose as a group close to each other (Figure 2). They were therefore clearly different from both *C. bovis *and *C. crewei*, and more similar to descriptions of mites *C. texanus*, *C. mydaus *and *C. panda*.

In comparison to male *C. texanus *isolated from cattle in Germany, the male *Chorioptes *from Swedish moose possessed longer opisthosomal setae 1 (seta *ae*), 2 (seta *l5*) and 3 (seta *d4*), while setae 4 and 5 did not differ significantly in length (table 1). The differences were most evident in seta 1 being 85 *μ*m long on average in the mites from moose, compared to an average length of 54 *μ*m in the *C. texanus *mites. This seta was also thicker in the mites from moose than in *C. texanus *(Figure 2). Additionally, the accessory opisthosomal lobe bearing seta 1 seemed to be smaller and proximally positioned in the mites from the moose (Figure 2).

### Molecular investigations

DNA sequences between primers RIB-3 and RIB-4, varying in length between 388 and 390 bp, were determined from ten clones originating from independent PCR amplifications using DNA extracted from skin scraping materials of moose M290. Taq polymerase errors and rare polymorphisms (which are indistinguishable), determined as individual mutations not found in any other sequenced clone, were detected at seven positions (six substitutions and one 1-base deletion) in seven of the ten clones, corresponding to a rate of 0.2%.

After excluding Taq polymerase errors and rare polymorphisms from further analysis, nine polymorphic positions, including seven substitutions as well as one 1-base and one 2-base insertion/deletion, were detected, resulting in eight different sequence types here called genotypes. Seven genotypes were found in the ten sequences of mites from moose M290, while two and three genotypes were obtained from mites collected from moose M289 and M291, respectively. Genotype 1 was the genotype detected most frequently, being found five times and in mites from all the three host animals. Genotype 2 included three sequences from moose M290 and M291, while two sequences were affiliated to genotype 3 from moose M289 and M290. All of the other genotypes were detected only once. The identities between the different genotypes varied from 97.5–99.7%. The sequences are available under the database accession numbers [GenBank:EF433564–EF433575].

**Figure 3:**
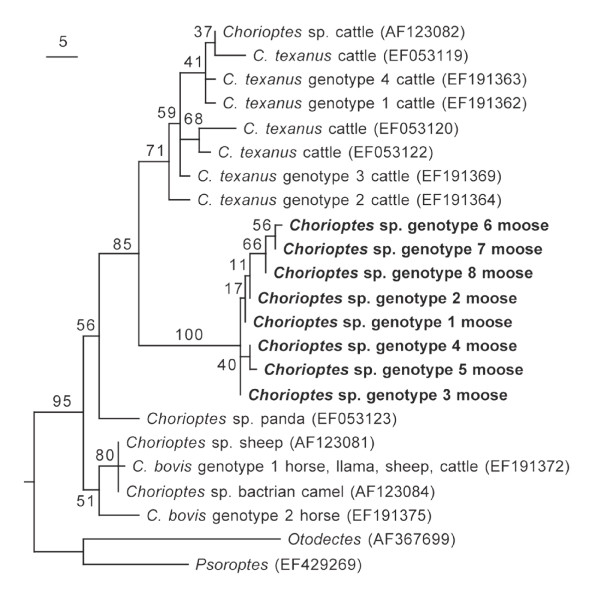
**Maximum-likelihood phylogenetic tree of *Chorioptes *spp. based on complete ITS-2 DNA sequences**. The corresponding sequences of *Psoroptes *and *Otodectes *were used as the outgroup (midpoint rooted). Bootstrap values are given as percentages for each node. The scale indicates the expected number of nucleotide substitutions. Database accession numbers of the sequences are given in brackets. The sequences from the *Chorioptes *mites from Swedish moose are in boldface.

The phylogenetic analyses produced a single maximum-likelihood tree (Figure 3) and 404 maximum-parsimony trees. The majority-rule consensus of the maximum-parsimony trees differed from the maximum-likelihood tree only in being less resolved; and the bootstrap support for the relevant branches was also approximately the same. The sequences of the *Chorioptes *mites from the moose formed a distinct monophyletic group in these trees, with a sister-group relationship to *C. texanus*. The support for the *C. bovis *group was poor, due to ambiguous support for the inclusion or exclusion of both genotype 2 and the *Chorioptes *taxon sampled from panda.

### Histopathology

**Figure 4:**
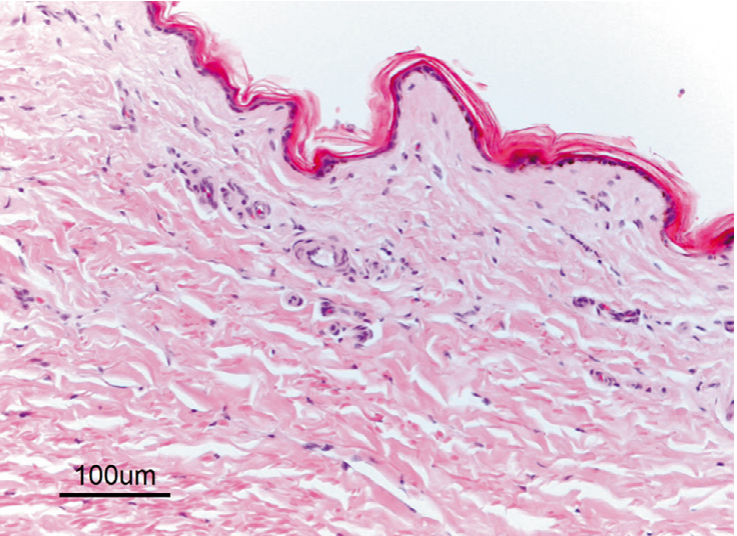
**Histology of normal skin from the outer ear canal in a moose not infested with *Chorioptes*. HE**. The epidermis is thin and the thickness of the stratum corneum exceeds that of cellular layers. Scattered lymphocytes in the superficial dermis.

In ears negative for *Chorioptes*, the epidermis consisted of 2–4 layers of epithelial cells. Over a single straight layer of basal cells were one or two layers of spinosum cells, mostly followed by one layer of granular cells. The thickness of the stratum corneum was somewhat variable, and occasionally exceeded the thickness of the cellular layers (Figure 4). In the superficial dermis were scattered T- and B-lymphocytes, plasma cells and occasional mast cells.

**Figure 5:**
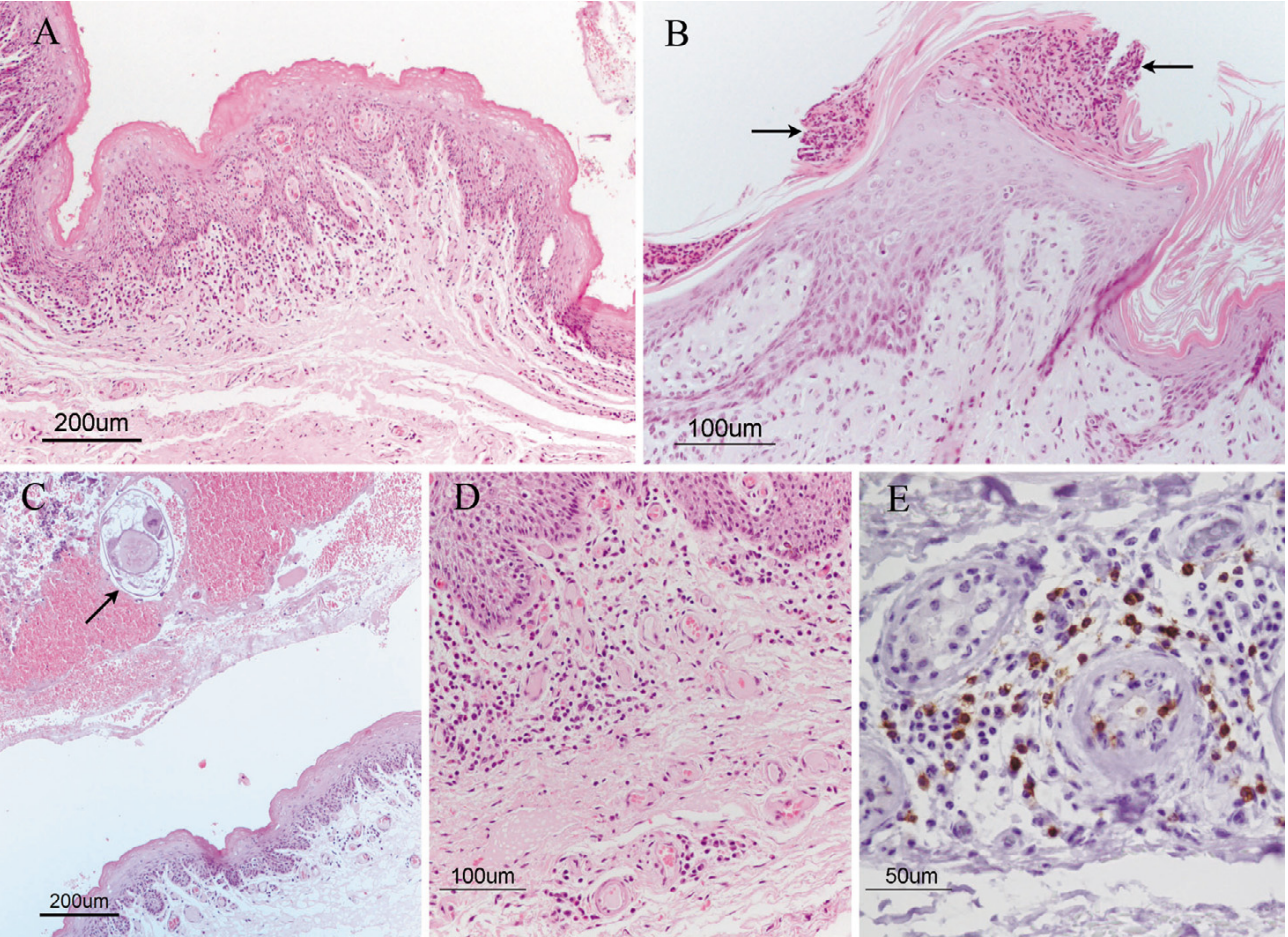
**Histopathology of the skin from the outer ear canal in a *Chorioptes*-infested moose**. **A: **Marked epidermal hyperplasia with prominent rete ridges (acanthosis) and orthokeratotic hyperkeratosis. HE. **B: **Acanthotic epidermis with multifocal crust formation (arrows) and mixed inflammation in dermis. HE. **C: **Haemorrhagic crust with embedded cross-sectioned mite (arrow), on epidermis. HE. **D: **Dermal inflammation, showing lymphocytes, plasma cells and some macrophages with a perivascular to interstitial distribution. HE. **E: **Immunohistochemistry for CD3 F7.2.38 (T-lymphocytes stained brown), showing perivascular infiltrate of T-lymphocytes and plasma cells in deeper parts of the dermis. En-Vision^+® ^with haematoxylin.

In ears with mites, the epidermis was in general moderately, and sometimes severely, hyperplastic. Mixed orthokeratotic and parakeratotic hyperkeratosis was a frequent feature, and in nine cases serocellular exudation with degenerated neutrophils and crust formation was seen (Figures 5A and 5B). Mild to moderate exocytosis of neutrophils into the epidermis occurred in seven cases. In 13 moose, intact mites or mite fragments were lying free on the surface among keratin fragments, or embedded in crusts (Figure 5C). Some cases showed mild to moderate spongiosis, and occasional vacuolated keratinocytes were found in the spinous and granular strata. Six moose had ulcerations in one or both ears, generally shallow, but occasionally deep. In four moose, ulcerations were acute, with neutrophil-rich serocellular exudates, and sometimes haemorrhages, whereas two moose showed chronic ulcers with fibrosis. In the dermis, mostly mild to moderate, but occasionally severe, perivascular to interstitial inflammatory infiltrates occurred consistently (Figures 5D and 5E). Superficial to mid-dermal infiltrates were as a rule dominated by T-cells and plasma cells, with an admixture of macrophages (not further characterized) and, in six moose, sparse eosinophils. Compared to non-infested ears, any increase of mast cells was not evident. Where inflammation extended to the deep dermis, the infiltrate was mainly perivascular and plasmacellular. Mild to moderate vascular congestion was common. Both ceruminal and sebaceous glands often showed mild to moderate hypertrophy, with the addition of moderate hyperplasia in ceruminal glands. All sections stained for fungi were negative. Small amounts of bacteria (gram-positive cocci) were found superficially on the skin surface or in crusts in a few ears.

### Microbiology

Among the 14 moose subjected to bacterial cultivation, *Staphylococcus aureus *was grown from five and *Staphylococcus *spp. from one moose, all mite-infested. Of the eight moose negative for bacterial culture, four were mite-infested. Yeasts, not further characterized, were cultured from two moose, both non-infested.

## Discussion

This study showed that *Chorioptes *sp. was frequently detected in the outer ear canals of moose (*Alces alces*) in Sweden. In wildlife, localization of *Chorioptes *mites in the outer ear canals has also been described in reindeer, *Rangifer tarandus *[7], red-flanked duiker, *Cephalophus rufilatus *[30] and giant panda, *Ailuropoda melanoleuca *[19]. In this study the skin of other parts of the body of the moose was not examined for mites and therefore it is not known if the *Chorioptes *infestation was restricted to the ear canals. Moreover, *Chorioptes *sp. have been found on alopecic areas of the skin in moose necropsied at SVA, and in one case *Chorioptes *were also demonstrated in the outer ear canals (pers. comm. C. Bröjer). However, these mites have not yet been closely compared to the *Chorioptes *sp. isolated from the external ear canals of the moose of this study.

The evidence presented here indicates that the *Chorioptes *sp. found is a previously undescribed species, which will be described in detail in a later publication. The *Chorioptes *sp. was closest to *C. texanus*, based on both morphology and genetics including cloning and sequencing of multiple independent PCR products from skin scrapings, in order to recognize different sequence types that might originate from different individual mites present in the skin, or from different copies of the rDNA which represents a multi-copy gene [5].

Morphological differentiation between *C. texanus *and *C. bovis *is mainly based on the setae at the opisthosomal lobes in male mites (Figure 2). The outer opisthosomal seta, called seta 1 [4] or seta *ae *[18], is much longer in *C. bovis *than in *C. texanus*, with a length >100 *μ*m and <100 *μ*m, respectively [5,7,31]. The mites from moose possessed shorter setae than those of *C. bovis*. Furthermore, they had longer opisthosomal setae 1, 2 and 3 compared to *C. texanus *mites from cattle. Whether the ear mites of reindeer, affiliated to *C. texanus *[7], might actually belong to the same species as the ear mites from moose of this study, can not be decided, since mites were not available for direct morphological and molecular comparison.

When the genotype sequences from the *Chorioptes *mites from moose were compared with sequences from *C. texanus *and *C. bovis *obtained using the same protocols [5], pairwise identities from the different *Chorioptes *moose genotypes were 89–91% (*C. texanus*) and 86–89% (*C. bovis*). These inter-species identities were thus of the same order as the identity of 89–93% found between *C. texanus *and *C. bovis *[5], for which separate species status is generally accepted. Furthermore, phylogenetic analyses (which were robust to the form of analysis used) indicated that the moose mites formed a monophyletic group, with a sister-group relationship to *C. texanus*. This separation is interpreted here as an indication of taxonomic separation and not as a reflection of the geographic origin of the mites, because the corresponding sequences of *C. texanus *and *C. bovis *grouped according to taxonomic entities and not the geographical origin of the mites, and the mites from Swedish moose did not group together with *Chorioptes *mites of their nearest geographical origin. Thus, there is no molecular support, either from genetic similarity or sister-group relationships, for placing the *Chorioptes *mites from Swedish moose in either *C. texanus *or *C. bovis*.

In the past, moose have been described as being parasitized by *C. texanus *in Poland [11,12], although neither pictures nor descriptions of the mites were given. These mites might be different from those that we found in moose in Sweden, because the mites in Poland were not found in the auricles of the animals but only at several other body sites [12]. We did not investigate non-auricular body sites in our study, leaving open the question as to whether other species of *Chorioptes*, such as *C. texanus*, are also found in moose in Sweden or if the *Chorioptes *sp. identified in this study affects other parts of the skin besides the ear canal.

The species status of the other three *Chorioptes *species seems doubtful [2]. *C. crewei *was based on morphological features of only four females and two males taken from the ears of a red-flanked duiker (*Cephalophus rufilatus*) in Cameroon [30,32], and detection of this species has never been documented afterwards. Mites of *C. mydaus *were isolated only once from a stink badger (*Mydaus lucifer*) in Borneo [18], and those of *C. panda *were found in the ears of giant pandas (*Ailuropoda melangolenca*) in zoos in France [19] and China [33]. Affiliation of the mites from Swedish moose with *C. mydaus *or *C. panda *was difficult to determine, since mites of these questionable species were not available for direct comparison. Morphological comparison was thus limited to published descriptions and drawings [18,19,33], which allowed only limited conclusions. The mites from the moose differed from *C. mydaus *by a shorter seta *ae*, which was 85 *μ*m on average compared to 100 *μ*m in *C. mydaus *[18]. Additionally, setae *l4 *and *d5 *were of a distinct flattened, blade-like shape in the mites from moose (Figure 2), while they were described and drawn to be small or only slightly widened in *C. mydaus *[18]. The moose mites were regarded as morphologically closer to *C. panda *based on drawings [19]. The most evident differences were that in *C. panda *seta *ae *was less separated by setae *l4*, *l5 *and *d5*, and all of these 4 setae arose at an oblique line from the opisthosomal lobe [19], while there was a distinct gap between seta *ae *and the other three seta mentioned in the mites from the moose, and the seta *ae *arose proximal to a line of the points of origin of setae *l4*, *l5 *and *d5 *(Figure 2). These differences, however, could not be detected in the drawings of *C. panda *by other authors [33].

No evidence was found to affiliate the *Chorioptes *sp. with any of these taxa. The sequence from mites sampled from panda in China [EMBL:EF053123], although not affiliated to a particular mite species, appears to exclude *C. panda *as a possible identification of the moose mite.

A high percentage of the moose (~81%) was shown to be *Chorioptes*-infested. Additionally the true prevalence of infestation might actually have been higher since in many cases the moose had been dead for several days, and the mites may had left the carcase. The direct ocular method employed, i.e. raising the temperature of the sampled pieces of ear canals, stimulated the live mites to migrate and thus facilitated their detection and collection. This method is able to detect live mites that are present in relatively high numbers. Detection of mites by skin scrapings, also performed in this study, is more frequently used [34], and demonstrates only a proportion of the mites actually present. Also, it does not distinguish live from dead mites. In addition, non-burrowing mites may occasionally be found in histological sections, however, most of the mites on the skin surface may be lost during the histological processing.

The majority of the moose were in a poor nutritional state, ranging from subnormal to cachectic. Mite-infested animals were more often recorded at necropsy as cachectic than were uninfested moose. However, possible associations between *Chorioptes *infestation and the nutritional state and other factors, including age, sex, season and concomitant diseases, were not analyzed because the sample was obtained from animals found dead in the field at different times of the year and sent for diagnostic post-mortem examination. This resulted in a great variability in host related factors and possible biases in sampling.

Since the samples were from wild animals, no observations on the duration of the infestations could be performed. The histopathological changes, however, suggested that most cases were subacute or chronic. Studies describing the pathology of chorioptic mange in various species, including ruminants, are few, and only concern mange caused by *C. bovis*. The epidermal changes seen in the moose, characterized by hyperplasia with ortho- and parakeratotic hyperkeratosis, sometimes with serocellular exudates and crusts, were similar to those described in sheep and a Japanese serow (*Capricornis crispus*) [35,36].

Eosinophils are common in allergic reactions, including those of ectoparasitic origin [37-39]. The inconsistent and sparse dermal tissue eosinophilia in the moose is in contrast to findings in *C. bovis*-infested cattle and horses, in which eosinophils were numerous [37,40], but are in accordance with observations in sheep with this mite [35].

In dermal allergic hypersensitivity reactions in domestic animals, a superficial perivascular distribution of inflammatory cells is commonly predominant [37,38]. Despite the type of hypersensitivity reaction, T-lymphocytes dominate, while B-lymphocytes and plasma cells are present in lesser amounts [38,39]. The dermal inflammation in the moose was characterized by perivascular to interstitial infiltrates of T-lymphocytes, plasma cells and B-lymphocytes, suggesting that also in the moose the reactivity may involve a hypersensitivity reaction. In *C. bovis*-infested sheep, macrophages, lymphocytes and plasma cells were found perivascularly in the superficial dermis, and the reaction was proposed to represent an allergic contact dermatitis [35].

Dermal infiltrates of plasma cells were prominent in the moose. Plasma cells are reported to be common in interstitial dermatitis in large domestic animals, and may be considered to be of low significance [37]. However, in dogs and cats, plasma cells are described as predominant in late stage pyoderma, but are less frequent in allergic hypersensitivity reactions [38]. A bacterial cause of the prominent plasma cell infiltration in the moose should be considered, because staphylococci, mainly *Staphylococcus aureus*, were cultured from the moose's ear skin in 6/10 infested ears, but in none of the non-infested moose. Nonetheless, in many species *S. aureus *belongs to the normal skin flora [41], and this could also be the case in the skin of the outer ear canals of moose. Irrespective if the ears were cultured positive for staphylococci or not, dermal infiltrates of plasma cells were a feature. Hence, an association between presence of plasma cells and positive culture could not be established. This might indicate another cause than bacterial infection to the plasma cell prominence.

## Conclusion

*Chorioptes *sp. was frequently detected in the outer ear canals of moose (*Alces alces*) in Sweden. This mite has morphological and genetic characteristics distinct from those of *C. texanus *and *C. bovis*, the two species generally accepted within the genus. Morphology also did not unequivocally argue for a diagnosis as *C. crewei*, *C. mydaus *or *C. panda*, and thus we argue that the mites belong to a proposed new species. The mites were obviously pathogenic to the moose, evoking epidermal and dermal inflammatory lesions, the latter indicating immunological hypersensitivity reactions.

## Competing interests

The author(s) declare that they have no competing interests.

## Authors' contributions

GH carried out the histopathological examinations, and wrote the first draft of the manuscript. MZR carried out the morphological and molecular studies, and wrote the first draft of the manuscript. DGW conducted the preliminary histopathology, collected samples, blocked the tissues and contributed to the manuscript. RL contributed to histopathological description and to the manuscript. RM carried out the bacteriological and mycological investigations. DM contributed to the data analyses, and revised and edited the manuscript. SB initiated and coordinated the study, collected the samples, carried out the parasitological investigations, and contributed to the manuscript. All authors read and approved the final manuscript.
